# Interannual climate variability improves niche estimates for ectothermic but not endothermic species

**DOI:** 10.1038/s41598-023-39637-x

**Published:** 2023-08-02

**Authors:** Dirk Nikolaus Karger, Bianca Saladin, Rafael O. Wüest, Catherine H. Graham, Damaris Zurell, Lidong Mo, Niklaus E. Zimmermann

**Affiliations:** 1 Swiss Federal Institute for Forest, Snow and Landscape Research WSL, Zürcherstrasse 111, 8903 Birmensdorf, Switzerland; 2 University of Potsdam, Maulbeerallee 3, 14469 Potsdam, Germany; 3 ETH Zurich, Universitätstrasse 16, 8092 Zürich, Switzerland

**Keywords:** Macroecology, Macroecology

## Abstract

Climate is an important limiting factor of species’ niches and it is therefore regularly included in ecological applications such as species distribution models (SDMs). Climate predictors are often used in the form of long-term mean values, yet many species experience wide climatic variation over their lifespan and within their geographical range which is unlikely captured by long-term means. Further, depending on their physiology, distinct groups of species cope with climate variability differently. Ectothermic species, which are directly dependent on the thermal environment are expected to show a different response to temporal or spatial variability in temperature than endothermic groups that can decouple their internal temperature from that of their surroundings. Here, we explore the degree to which spatial variability and long-term temporal variability in temperature and precipitation change niche estimates for ectothermic (730 amphibian, 1276 reptile), and endothermic (1961 mammal) species globally. We use three different species distribution modelling (SDM) algorithms to quantify the effect of spatial and temporal climate variability, based on global range maps of all species and climate data from 1979 to 2013. All SDMs were cross-validated and accessed for their performance using the Area under the Curve (AUC) and the True Skill Statistic (TSS). The mean performance of SDMs using only climatic means as predictors was TSS = 0.71 and AUC = 0.90. The inclusion of spatial variability offers a significant gain in SDM performance (mean TSS = 0.74, mean AUC = 0.92), as does the inclusion of temporal variability (mean TSS = 0.80, mean AUC = 0.94). Including both spatial and temporal variability in SDMs shows the highest scores in AUC and TSS. Accounting for temporal rather than spatial variability in climate improved the SDM prediction especially in ectotherm groups such as amphibians and reptiles, while for endothermic mammals no such improvement was observed. These results indicate that including long term climate interannual climate variability into niche estimations matters most for ectothermic species that cannot decouple their physiology from the surrounding environment as endothermic species can.

## Introduction

Climate has long been considered among the strongest determinants of species distributions, often imposing physiological limits on where a species can occur^1^. Climate can influence species’ distributions^2^ at a variety of temporal and spatial scales. For instance, distributional ranges can be limited by annual seasonal variation in temperature or precipitation^3^ or by climate fluctuations acting over hourly to daily time steps^4,5^. From a spatial perspective, species distributions can be influenced by both general climatic conditions characteristic of a given region, as well as micro-climatic conditions afforded by structure in a landscape, such as the shade of a tree, or underside of a rock^6^. As a result, detecting the impact of climate on species distributions will depend, to a large degree, on the temporal and spatial scale considered^7,8^. Yet, while the importance of scale has long been acknowledged as a key driver of species’ range limits, the major approach to mapping ranges, namely species distribution models (SDMs), uses exclusively climate summarized over time and gridded at specific spatial grains. SDM studies that consider multiple spatial and temporal scales reveal improved SDMs, but this multi-scale approach has only been applied to a limited number of ecologically well-known species^9,10^. Here, we build on these promising, yet only marginally explored, results by evaluating the performance of SDMs when built considering both temporal and spatial climate variations for 3967 species that vary in their geographic distribution, and ecological and physiological requirements.

SDMs, sometimes also termed bioclimatic envelope or habitat suitability models^11,12^ characterize the environmental niche of a species^13^ usually based on a few key environmental factors, such as temperature and precipitation. This, often limited, set of key variables is generally^14^ derived from long-term means (climatologies) alone^15^. Such aggregations of climate variability to long-term means remove however, information on the temporal inter-annual or even daily to hourly variability that might influence species distributions. Spatial climate heterogeneity resulting from small-scale topography and other factors is neither represented in coarser-scale gridded datasets, yet it can be essential for quantifying species’ environmental niches^16,17^.

Species often experience a wide range of climatic variation over their lifespan and within their geographical range. However, they can strongly differ in the degree to which they can tolerate climatic variability^18^. A species’ ability to cope with climate variability will thus influence their spatial distribution. For instance, endotherms and ectotherms cope with climate variability quite differently. By generating their own heat, endotherms have a greater capability to buffer the influence of climate variability than ectotherms which rely on behavioral modifications or micro-habitat use to maintain their body temperatures and buffer climate variation. The different ways species respond to climate variability might be of increasing concern since one of the major components of climate change is an increase in climate variability and – as a consequence – an increase of extremes^19,20^. Increasing climatic variability may therefore cause greater physiological stress to ectotherms compared to endotherms with likely severe consequences for their spatial distribution^21,22^.

The influence of climate variability on species might also depend on the geographic regions in which they occur. In the tropics, species generally experience a lower degree of intra- and interannual climatic variation due to the rather stable environmental conditions they encounter throughout the year^7,23^. In temperate climates however, the conditions a species experiences are much more variable due to the larger intra- and interannual variation in climate^23^. Species occurring in tropical ecosystems, therefore often have a much narrower climatic niche than in temperate regions^24,25^. In turn, this implies that the influence of temporal variability might be greater in areas where species are not well adapted to variation in climate, i.e. the tropics. Yet, despite the difference between the tropics and temperate climates, interannual variation in climate can pose physiological constraints on species, e.g. a year with poor conditions can have an effect on the reproductive success in the following year as shown for passerine birds in Europe^26^. Interannual climate variation is also known to affect the activity and thermoregulation of lizards as their physiology is sensitive to too cold^27^, or too warm^28^ conditions that are outside their thermal optimum. Such species may prefer low inter-annual variation, while species with broader thermal tolerances might gain a competitive advantage in areas with higher interannual variation in temperatures.

A misrepresentation of spatial heterogeneity when representing or aggregating climate predictors at coarse grain might also more strongly impact niche estimates in the tropics compared to temperate or boreal zones^23^.

Therefore, along large-scale geographic gradients both spatial and temporal variability can become important in estimations of a species environmental niche.

Here, we evaluate the performance of SDMs when they consider both temporal and spatial variation in estimating environmental niches for 2006 ectothermic species (730 amphibian, 1276 reptile) and 1961 endothermic (mammal) species. We hypothesize that:

- **H1:** The inclusion of spatial and temporal variability will positively affect the performance of SDMs,
- **H2:** The performance of ectotherm SDMs will improve more when accounting for spatial and temporal variability than the performance of endotherm SDMs, and
- **H3:** SDMs for tropical and mountain species will benefit more from including variability than SDMs of species from other habitats.

We test these hypotheses by modelling the global distribution of all species as a function of current climate at a 0.5° spatial resolution using four different predictor groups composed of different combinations of input variables: mean climatic conditions, spatial climatic variability and temporal (interannual) climatic variability.

## Results

### Spatial patterns of the climate predictors

**Figure 1:**
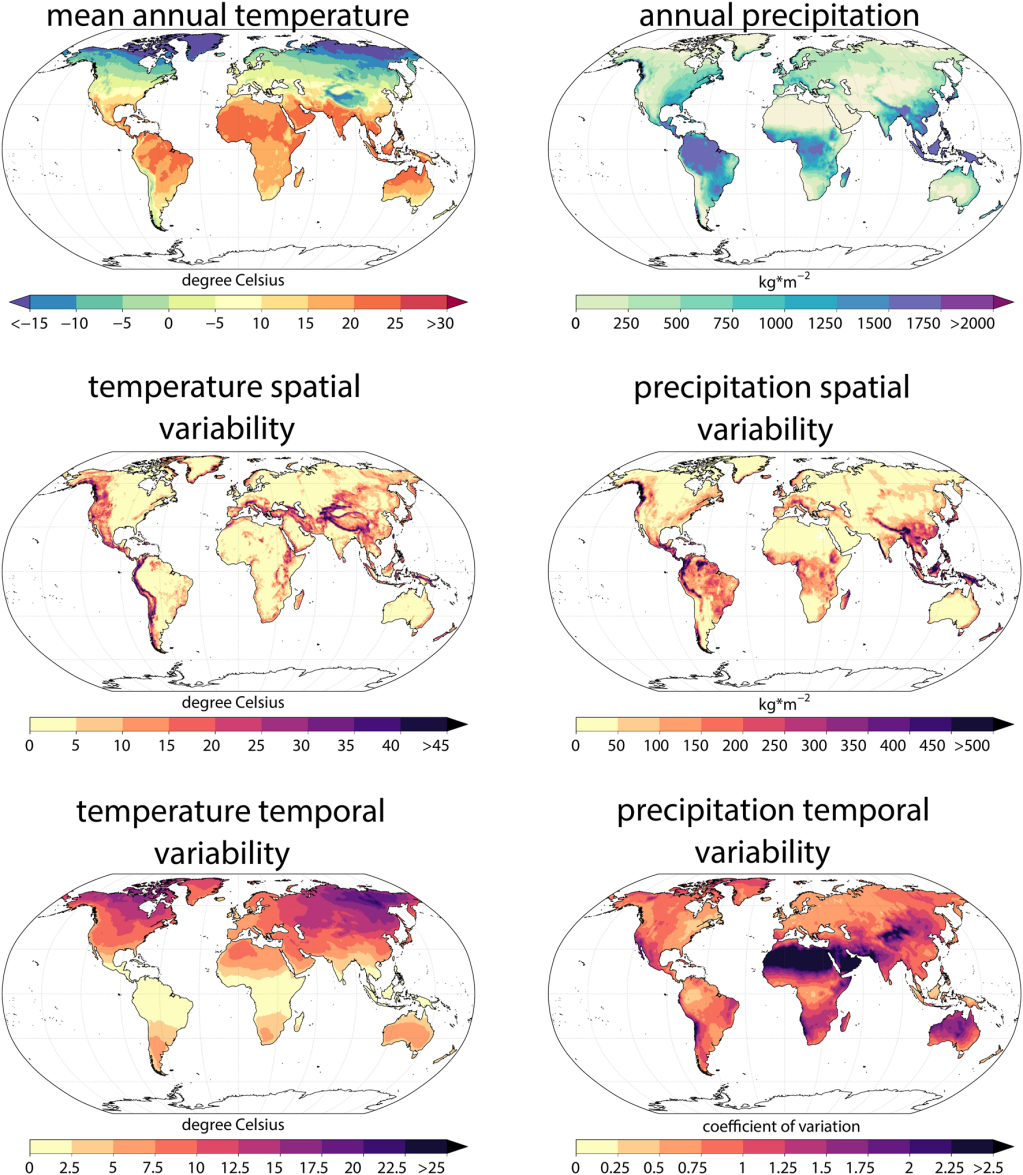
Spatio-temporal variation in 2 m air temperature and precipitation used as predictors in the different SDMs based on CHELSA V1.2. Mean annual values (upper row) show the annual mean for temperature, and the mean annual sum for precipitation averaged over the years 1979–2013 and aggregated to 0.5° from a 30 arc second spatial grain by taking the mean of a 0.5° grid cell. Spatial variation (middle) indicates the standard deviation (SD) of all temperature values of a 30 arc second grid of temperature or precipitation overlapping with a 0.5° grid cell. Temporal variation (lower row) shows the standard deviation (SD) of temperature over years between 1979 and 2013, and the relative standard deviation (RSD) calculated as the coefficient of variation for precipitation over the same time period.

While mean annual 2m air temperatures and annual precipitation sums were generally higher in the tropics and decreased towards the poles (Fig. 1, upper), the spatial variability (spatial SD, the standard deviation across all 30 arc second grid cells within the target grid size of 0.5°) of these two variables is usually highest in mountainous terrain (Fig. 1, middle). Interannual variability of temperature is generally higher in the northern hemisphere compared to the southern hemisphere (Fig. 1, lower), and increases from tropics to continental artic or boreal areas. Interannual variability of precipitation is more idiosyncratic, with lowest values estimated in desert areas.

### Performance scores of the SDMs with different predictors for the different taxa

Overall, the predictive performance of the SDMs was high with an average AUC of 0.92 and ranging from 0.90 to 0.95 between different groups of predictors and different SDM algorithms. For TSS, the average value was 0.75 with a minimum 0.68 and a maximum of 0.82 across different groups of predictors and different SDM algorithms (generalized additive models, GAM; generalized linear models GLM; random forests, RF). SDMs based only on long-term mean climate predictors performed worst among all groups, with average AUC and TSS scores of 0.90 and 0.71, respectively (GAM: 0.91; 0.73, GLM: 0.90; 0.73, RF: 0.90; 0.68). SDMs based on long-term mean climate predictors plus spatial variability performed slightly better, with average AUC and TSS scores of 0.92 and 0.74 (GAM: 0.93; 0.77, GLM: 0.92; 0.76, RF: 0.91; 0.70). SDMs based on mean climate predictors plus temporal variability performed better still, with average AUC and TSS scores of 0.94 and 0.80 (GAM: 0.94; 0.81, GLM: 0.94; 0.81, RF: 0.94; 0.77). SDMs containing mean climate predictors plus spatial and temporal variability showed average AUC and TSS scores of 0.94 and 0.80 (GAM: 0.95; 0.82, GLM: 0.93; 0.81, RF: 0.94; 0.77).

**Figure 2:**
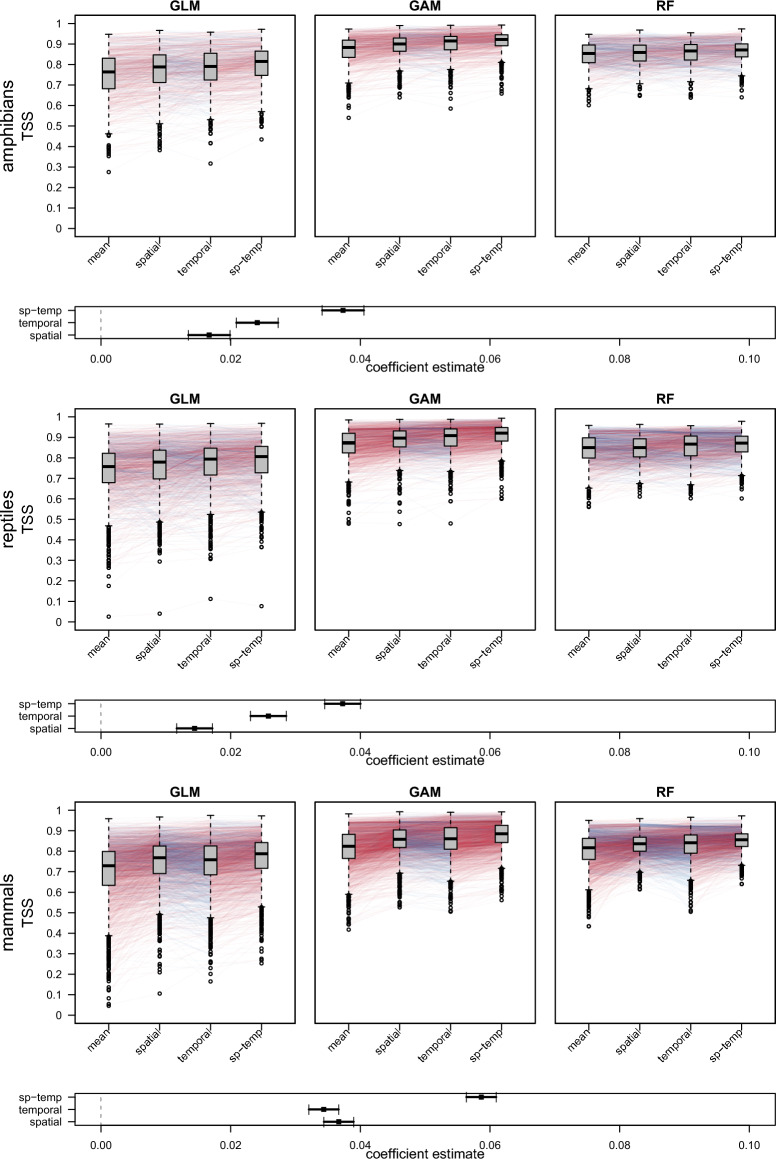
Comparison of the performance of three different SDM algorithms (*GLM* generalized linear model, *GAM* generalized additive model, *RF* random forests) calculated with four different sets of predictors for amphibians, reptiles, and mammals measured by the True Skill Statistic (TSS). Colored lines connect pairs of SDMs based on different predictor sets for the same species, with red and blue lines indicating pairs in which TSS values increased and decreased between predictor groups from left to right. Plots below the boxplots shows the coefficient estimates of a linear mixed effects model with TSS as response, the groups (mean, spatial, temporal, sp-temp) as predictor, and the model (GLM, GAM, RF) as well as the species ID as random effects. Coefficients are in relation to the performance of SDMs with the predictor set: mean.

A linear mixed effects model to assess the SDM performance with different predictor groups revealed that adding predictors that account for either spatial or temporal variability increased predictive performance of models across all vertebrate taxa. Models including temporal variability outperformed models including spatial variability for amphibians and reptiles, while performance of these models was equal for mammals. Models including both spatial and temporal variation performed best across all taxa. Fig. 2 illustrates these results using effect-size plots (below boxplots) for TSS; the results for AUC are equivalent (see Supplemental Fig. S1).

### Geographic differences

**Figure 3:**
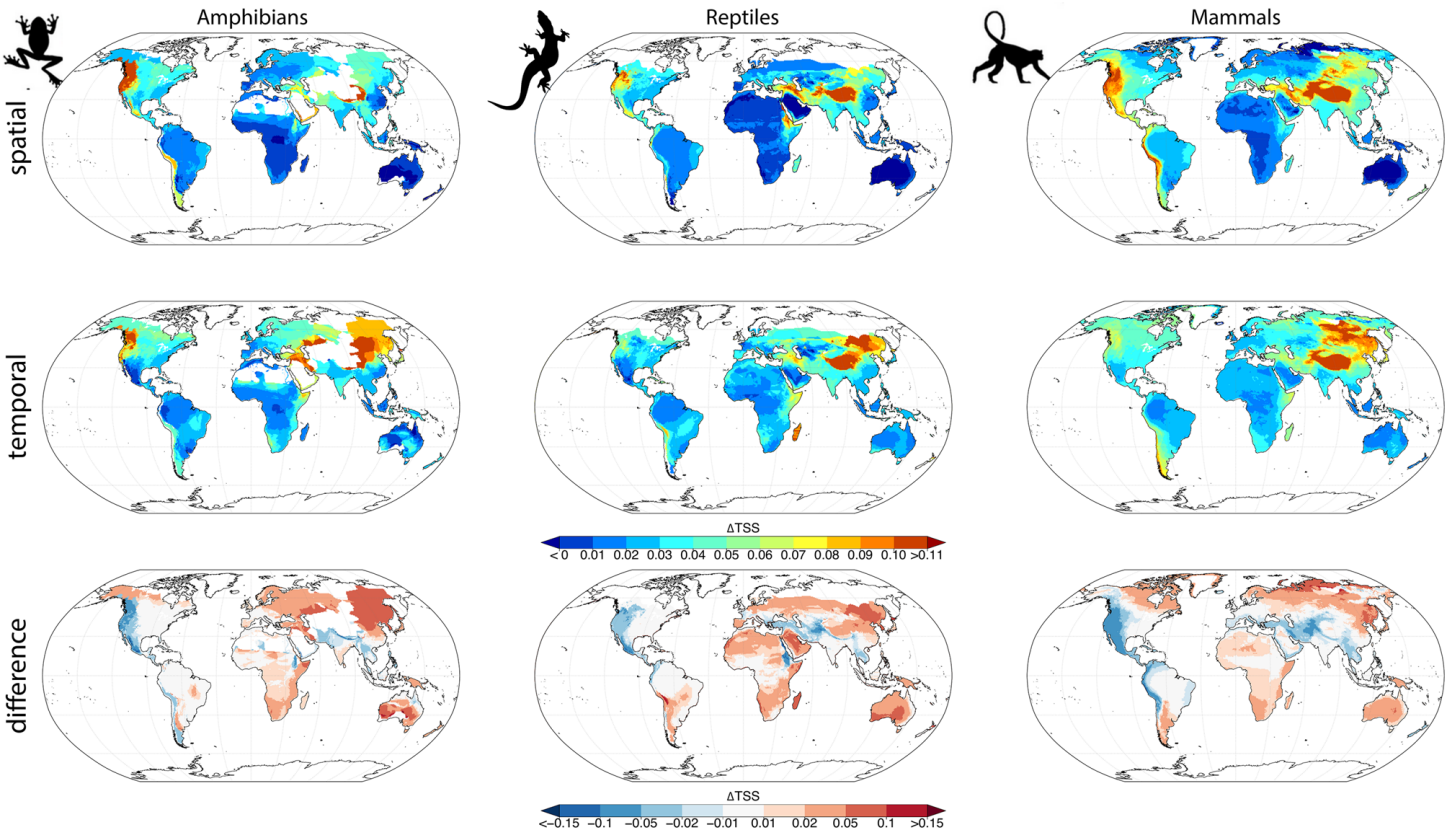
Comparison of the unique contributions in TSS of either the spatial (upper row), or temporal (interannual variability) predictors compared to mean climate as predictor, for all three taxonomic groups. The bottom row shows the differences in performance gain between spatial and temporal predictors, with blue indicating that accounting for spatial variability outperforms models that account for temporal variability, and red indicating that temporal outperforms spatial, indicating where each of the predictor types matters more. To test if different predictor groups have different performances in different regions, we used the gridded range map at 0.5° resolution from IUCN and assigned the value of the respective test metric (TSS, AUC) to the entire range in which a species is present. All ranges were then stacked and the mean of all TSS and AUC values covering a 0.5° grid cell calculated and differences (ΔTSS) have been calculated pairwise per species and then averaged over all species.

The performance of SDMs is highly variable across the globe (Fig. 3). The mean predictor group performed worst in mountainous terrain, such as the Andes or the Himalayas, but also Madagascar showed low TSS and AUC scores (Supplementary Figs. S1, S2 for AUC). Including spatial variability in temperature and precipitation in SDMs improved the models in these areas but showed a slight decline in performance in desert and arctic areas (Fig. 3: spatial). Adding temporal variability to the models containing mean predictors resulted in improved SDM performance in almost all areas (Fig. 3: temporal). Including both spatial and temporal variability resulted in a slight improvement as compared to models that included only spatial or temporal variability alone (Supplementary Fig. S1: spatio temporal).

The unique changes in TSS that are due to including either the spatial or temporal variability in addition to long-term means vary not just by taxon, but also across geographic regions (Fig. 3). For the ectothermic groups, amphibians and reptiles, the temporal predictor group increases the TSS in almost all areas except the western mountainous areas of the Americas, as well as the Himalayas, where the spatial predictor is still more important (Fig. 3). For mammals, the spatial predictor is much more important in mountainous terrain, while the temporal predictor only increases TSS in less heterogeneous terrain (Fig. 3). Latitudinal trends were specifically pronounced in Amphibians, were the inclusion of variability increases the performance of SDMs especially in higher latitudes (Supplementary Fig. 5). For mammals only the mid latitudes shows a higher performance than the average, while for reptiles no latitudinal trend emerged (Supplementary Fig. 5).

## Discussion

While SDM performance increases for all taxa when including climate variability additionally to long-term means, confirming our first hypothesis, this increase is not equal across the three taxonomic groups analyzed here. Although both spatial and temporal variability matter for all groups, the effect of temporal variability seems much larger for the ectothermic groups compared to the endothermic mammals.

The two ectothermic groups (amphibians and reptiles) show a significantly higher SDM performance when temporal climate variability is included over the case when only spatial variability is included. SDMs for the endothermic mammals do not differ significantly when either spatial or temporal variability are added to the mean climate.

This difference between groups might be explained by the differences in physiology between these groups consistent with our second hypothesis. All three groups have evolved differently in response to their environment, with ectothermic groups being much less adaptable to climatic variations than endothermic groups^29^. Such evolved differences in physiology ultimately affect how organisms interact with and are constrained by their environment^30^. Ectothermic species for example cannot buffer climate variation as well as endothermic species^31–34^ which have evolved the physiological capacity to regulate temperatures to some extent^35^. When building SDMs from long-term climatic means alone, thus neglecting the interannual dimension of climate, we miss out on important constraints especially for ectothermic species distributions, ultimately limiting the accuracy of niche estimations^36^.

Model formulation and parametrization certainly plays a role in the observed differences between predictor groups. More predictors in a model usually lead to a better overall fit of a model^37^ which can partly explain the increase in predictive power when the predictors based on mean climate are complemented with either spatial or temporal variability. However, as both the spatial and temporal predictor groups have the same number of variables, this effect does not apply when comparing TSS or AUC values resulting from these two predictor groups. Also, we observed both increasing and decreasing performance when including spatial or temporal variability compared to SDM including mean climate only (Fig. 3), meaning that the number of predictors alone could not satisfactorily explain the observed differences. Combining mean with spatial and temporal variability lead to an additional improvement. Yet, at this point, the parametrization of the model has not plateaued and model performance still increases when using both spatial and temporal variability as predictors^37–40^. Using different SDM algorithms affects the absolute performance of the SDMs in terms of the specific test metric (AUC, TSS). However, it did not affect the relative difference in model performance between SDMs calculated from different predictor groups, confirming that the observed differences are robust towards algorithm selection.

Including temporal variability of predictors into broad-scale SDMs leads to a greater improvement of model performance than the inclusion of sub-grain spatial variability of these predictors. These findings suggest that especially the inclusion of interannual climate variability has a large potential of improving the estimation of niche characteristics across a broad range of taxa, and especially narrow range species. Including temporal predictor variability increased the performance of SDMs in almost all areas across the globe, though to differing degrees and differently for the three groups at different latitudes. Especially prominent is the increase in areas with marked seasonality such as in tropical monsoon climates, tropical wet and dry climate, or areas that receive very infrequent precipitation such as the Horn of Africa^41^. Temporal variability does also increase the performance of SDMs in mountainous regions potentially indicating that temporal variability in a climate variable is also capable to capture the niche limitations that are otherwise captured by spatial variability. One reason for the performance gain when including temporal variability, especially interannual variability, is that it expresses the degree of climatic extremes which can physiologically limit the distribution of species^36^. Although, the degree to which extremes are represented in such variability predictors certainly depends on the temporal resolution of the climatic input dataset. In the case presented here, we used interannual variation, which means that extreme events are restricted to extremely dry or wet years, or extremely hot or cold years. Using more detailed temporal analyses would allow us to refine the representation of climatic extremes further.

As expected, the inclusion of the spatial variability improves SDMs mainly in mountainous areas where climate is extremely heterogeneous over short distances. The improvement was specifically strong in tropical mountains where species usually occur in narrow elevational bands with little or no intra-annual variability^23,42^. This improvement especially for narrow range species is also visible when predicted vs. observed range sizes are compared after inclusion of either spatial, temporal, or spatio-temporal variability into SDMs (Supplementary Fig. S5). In topographically less heterogenous terrain however, we observed a decline in the predictive power of SDMs. Almost all over Africa, Australia, and the low elevation parts of Eurasia and North America spatial variability has no effect, or even a negative effect on the performance of SDMs. In these areas spatial heterogeneity is low and inclusion of spatial variability in climate predictors seems biologically unimportant, which leads to a decrease in their performance^43–46^.

Our SDMs were calibrated using distribution data from expert range maps for the three taxonomic groups studied. This may lead to a bias compared to the use of exact point locations (such as those available in GBIF and other global databases), as distribution maps are not very accurate in terms of the geographical location of species observations. Their advantage lies in the almost complete coverage of the range, while their disadvantage lies in the difficulty of determining more precisely where a species is present or absent. Range polygons are generally not drawn with great precision and may therefore cover small regions of absence (and therefore contain false occurrences) or fail to distinguish small outliers of presence (and therefore contain false absences). In contrast, the approximate extent of occurrence is well documented and the main regions of absence are also well mapped. Therefore, models based on range maps should not be analyzed with a spatial resolution that is too fine for the precision with which the polygons were drawn. Previous studies have shown that a resolution of 0.5° to 1.0° is appropriate^47,48^. Point-based observations, such as those available in GBIF, have the advantage of allowing more accurate estimates of the niche characteristics of a species, as they are often available with a geographical precision of 1 km or finer. This allows full use of modern climate and environmental data such as CHELSA^49–51^ for SDM modelling. This is an advantage over polygon-based distribution data, as niches can be calibrated more precisely. However, such data have a major drawback: the sample selection is biased and information on absences is missing. Firstly, most GBIF sample points today come from citizen scientists, and they can submit (e.g. via iNaturalist and other portals) any observations they find^52,53^. Therefore, observations can include specimens from the natural range of a species, but also from private or botanical gardens, or from the invaded range (in the case of invasive species) of a species. Furthermore, the distribution of sampling sites does not follow a statistical design. Some sites are heavily oversampled, while large areas are completely undersampled^54,55^. In addition, the precision of geolocation is not always clear, and may partly be huge, despite having many significant digits on coordinates^52^. These problems occur because the data originate from a plethora of different sampling efforts and many of these do not carefully consider the major issues around sampling distributions (even sampling in space, accurate information on geolocation and its uncertainty). Considerable efforts are therefore needed, species by species, to postprocess species distribution data from such databases before it can be used^56,57^, or to combine the two differing data sources^58^. Since we analyzed our large species groups only at a comparably coarse spatial grain of 0.5°, we considered the use of range polygons as appropriate

With an increasing need in biodiversity modeling for current, past, and future predictions a better understanding of the climatic predictors that quantify the ecological niche of a species is needed. Here, we show that specifically the inclusion of temporal variability offers a promising improvement in modelling the current distribution of species. Yet, also the inclusion of spatial (sub-grain) variabilities can improve model accuracies, primarily in mountains and most clearly in tropical mountains. In summary, we anticipate that a more detailed inclusion of interannual climate variability offers a highly promising avenue for improving species distribution modelling in the future for groups that have a strong connection to the environment such as ectotherms.

## Methods

### Species data

We used global distribution maps provided by the Amphibian, Mammal, and Reptile Red List Assessment^59^. Grid cells within the distribution range of each species were converted to 0.5° grid cells, which is close to 50 km at the equator, a resolution suggested as appropriate^47^ and often used^60–62^ when gridding polygon range maps at the global scale. Grid cells intersecting with a range map polygon were assigned as presence cells, while those not intersecting were treated as absence cells. We only considered species for which the presences cover at least 72 0.5° grid cells so that a minimum of six data points per predictor variable (including quadratic terms) was available for model building. We also removed domestic and aquatic species.

### Climate predictor groups

**Figure 4:**
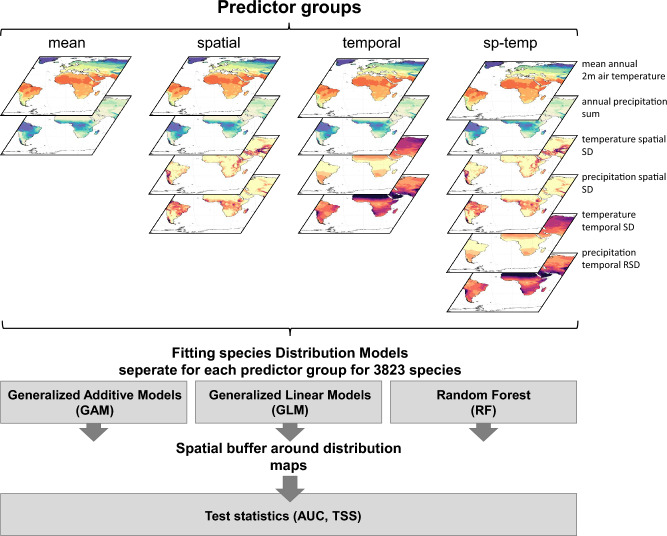
Schematic representation of the analytical setup. Four predictor groups were formed and three algorithms for species distribution models (SDMs) were fitted from range maps for 3967 species of mammals, amphibians, and reptiles. The different SDMs were predicted spatially, and their predictive performance assessed within a buffer of 3000 km around observed ranges, using the area under the curve (AUC) and the true skill statistic (TSS) as performance measures.

We used global climate data from CHELSA V1.2^49,63^ and built several groups of predictors (Fig. 4, Table 1) by aggregating CHELSA V1.2 to the 0.5° grid of the species data by taking the mean of all 30 arc second grid cells overlapping with a 0.5° grid cell. To calculate sub-grid heterogeneity of a climatic variable (hereafter: spatial) within a 0.5° grid cell, we used the standard deviation of all CHELSA 30 arc second grid cells overlapping with 0.5° grid cells. To calculate the interannual variability (hereafter: temporal) we calculated the standard deviation of mean annual 2m air temperature for each year from 1979 to 2013 from CHELSA V1.2 per grid cell. For temporal precipitation variability we used the relative standard deviation (temporal RSD, equivalent to the coefficient of variation) of the annual precipitation sum across all years from 1979 to 2013 from CHELSA V1.2 per grid cell. Based on the data aggregated as explained above, we generated four different groups of predictors for annual temperature and precipitation, with different combinations of spatial and temporal variabilities (Table 1).

**Table 1:** Characterization of the four predictor groups used with the respective variables included in each group as well as the temporal or spatial unit over which they were aggregated.

Predictor group	Variables included	Aggregation unit
Mean	Mean annual 2 m air temperature 1979–2013	–
	Mean annual precipitation sum 1979–2013	–
Spatial	Mean annual 2 m air temperature 1979–2013	–
	Mean annual precipitation sum 1979–2013	–
	Standard deviation mean annual 2 m air temperature 1979–2013	All 0.0083334° grid cells within a 0.5° grid cell
	Standard deviation mean annual precipitation sum 1979–2013	All 0.0083334° grid cells within a 0.5° grid cell
Temporal	Mean annual 2 m air temperature 1979–2013	–
	Mean annual precipitation sum 1979–2013	–
	Standard deviation mean annual 2 m air temperature 1979–2013	All years from 1979–2013
	Coefficient of variation mean annual precipitation sum 1979–2013	All years from 1979–2013
sp-temp	Mean annual 2 m air temperature 1979–2013	–
	Mean annual precipitation sum 1979–2013	–
	Standard deviation mean annual 2 m air temperature 1979–2013	All 0.0083334° grid cells within a 0.5° grid cell
	Standard deviation mean annual precipitation sum 1979–2013	All 0.0083334° grid cells within a 0.5° grid cell
	Standard deviation mean annual 2 m air temperature 1979–2013	All years from 1979–2013
	Coefficient of variation mean annual precipitation sum 1979–2013	All years from 1979–2013

All variables are based on CHELSA V1.2^49^.

### Species distribution modelling

We used three algorithms to relate presences and absences with the selected environmental predictor sets: generalized linear models (GLM)^64^, generalized additive models (GAM)^65^, and random forests (RF)^66^. GLMs were run using linear and quadratic terms, GAMs were run using thin plate splines setting an upper limit of 4 degrees of freedom (k = 5). In both cases, we set weights such that the sum of weights of presences equaled the sum of weights of absences^67^. A classification RF was fitted using 1500 trees, while sub-sampling was restricted to contain equal numbers of presences and absences.

To assess model performance, we tested SDM predictions only within a buffer around each species’ range polygon. By doing so we account for biogeographic history and explicitly test how well a model predicts the actual range of a species rather than how well it also makes predictions in regions far outside a species’ range, yet with suitable climate. We applied a buffer of 3000 km around each range polygon and fitted and tested SDMs only within this extent.

As absences we used all grid cells within the 3000 km buffer around each IUCN range map that do not overlap with a 0.5° grid cell, as presences all cells that overlap with a 0.5° grid cell. Therefore the SDMs are a combination of environmental suitability and dispersal constraints to better estimate the distribution of a species. The use of 3000 km is a compromise to balance between buffering presences such that absences sampled within the buffer are meaningful as the buffer is large enough to constrain distribution models accurately between suitable and unsuitable habitats, and small enough to avoid excessive overprediction of species’ distributions. The choice of the buffer size is also due to the coarse resolution of the grids (0.5°). A very small buffer would hardly leave any absences in this case, while a too large buffer might inflate the performance statistics by sampling absences anywhere far away from species occurrences. Additionally, it also enhances the discrimination between presence and absence observations as sufficient absences are sampled nearby to presences^68,69^. The number of presences vs. absences using this approach, can however, be quite imbalanced, with small range species having more absences, and large range species less absences in this case. To counterweight this imbalance in presences vs. absences we used an inverse weighting of the presences and absences so that the sum of the absences equals the sum of the presences. We used a World Azimuthal Equidistant (EPSG: 9001) projection for the buffer.

We evaluated the predictive performance of the SDMs using repeated split-sample tests: we split the data repeatedly into 80% training and 20% test data, fitted the model on the training data, and predicted it to the test data. This procedure was repeated 30 times, while we recorded predictive performance of each repeat. Each split was generated such that 80% of presences and absences were generated independently, thus ascertaining that each test was done with the same prevalence. By doing this, the individual repeats are always constructed the same way and do not vary in overlap. Predictive performance was assessed using a) the true skills statistic (TSS)^70^, after thresholding the predictions into presence/absence using a TSS-optimized threshold, and b) the area under the curve (AUC)^71^. We provide the full SDM description following the ODMAP protocol^72^ in Supplementary Table S1.

### Performance tests of predictor groups

We used a linear mixed effects model^73^ with either TSS or AUC as response variable and the predictor group as fixed effects together with the SDM algorithm (GLM, GAM, RF) and the species identity as random effects. Adding the type of SDM (GLM, GAM, or RF) as random effect on the intercept considers that algorithms can perform differently well (e.g. have a different mean performance between AUC or TSS)^62^. To always compare the same species, but modeled with different sets of predictor groups, we also added the identity of the species as random affect to the intercept.

All analyses have been performed using the R language for statistical computing^74^, the R packages raster^75^, mgcv^76^, and randomForest^77^.

### Geographic comparison of the performance of different predictor groups

To test if different predictor groups have different performances in different regions, we used the gridded range map at 0.5° resolution from IUCN and assigned the value of the respective test metric (TSS, AUC) to the entire range in which a species is present. All ranges were then stacked and the mean of all TSS and AUC values covering a 0.5° grid cell calculated.

Additionally, we calculated the observed range size defined as the number of pixels occupied and compared it to the predicted range size within the 3000 km buffer defined as the numbers of pixels occupied after thresholding the probability estimates from the SDMs. The observed vs. the predicted range size has then been compared by calculating the mean squared deviation.

To detect latitudinal trends in the improvement of SDMs after inclusion of either spatial, temporal, or spatio-temporal variability in SDMs, we calculated the mean and standard deviation of the difference between TSS values derived from SDMs only including mean climate, with either spatial, temporal, or spatio-temporal derived TSS values in 1° latitudinal bands.

## Supplementary Information

Supplementary Information.

## Data Availability

The data that support the findings of this study are openly available in EnviDat (envidat.ch) at https://www.doi.org/10.16904/envidat.354. Codes related to this study are available here: https://gitlabext.wsl.ch/karger/sdms_climate_variability.
