# Supplementary material for: Interannual climate variability improves niche estimates for ectothermic but not endothermic species
Source: Sci Rep. 2023 Aug 2;13:12538. doi: 10.1038/s41598-023-39637-x (PMC10397316; doi:10.1038/s41598-023-39637-x)
Supplement: Supplementary file 1 — Supplementary Information. [file 41598_2023_39637_MOESM1_ESM.pdf]

Interannual climate variability improves niche estimates for ectothermic but not endothermic species

Dirk Nikolaus Karger[1,*], Bianca Saladin[1], Rafael O. Wüest[1], Catherine H. Graham[1], Damaris Zurell[1,2], Lidong Mo[1,3], and Niklaus E. Zimmermann[1]

[1]Swiss Federal Institute for Forest, Snow and Landscape Research WSL, Zürcherstrasse 111, 8903 Birmensdorf, Switzerland
[2]University of Potsdam, Maulbeerallee 3, 14469 Potsdam, Germany
[3]ETH Zurich, Universitätstrasse 16, 8092 Zürich, Switzerland
[*]dirk.karger@wsl.ch

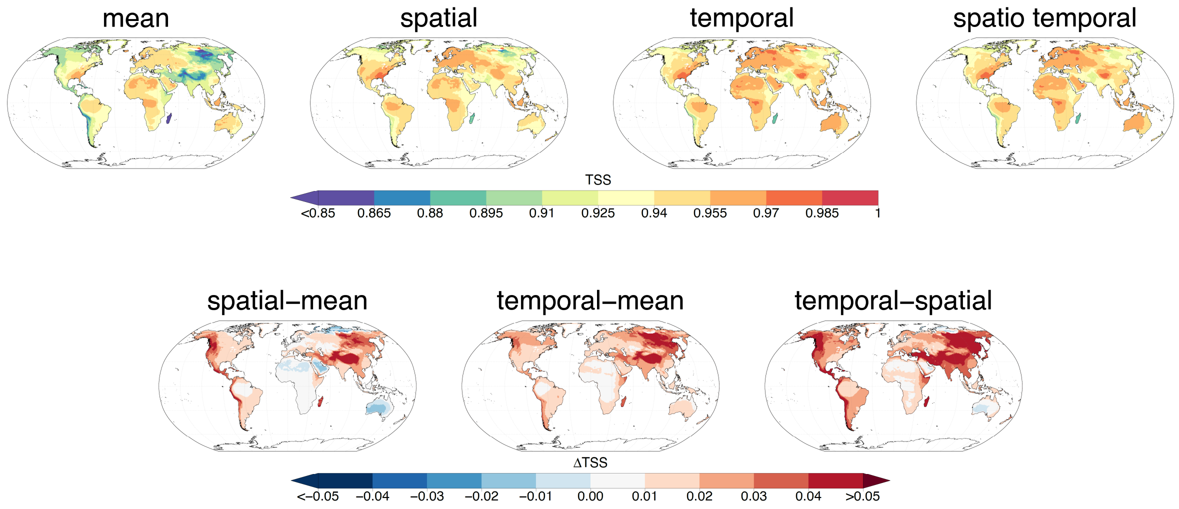

Supplementary Figure S1 Spatial variation in mean TSS (True Skill Statistic) values per grid cell and TSS differences between models using different predictor groups. The upper row illustrates TSS averaged for all mammals, reptiles, and amphibians modeled for the four models using different predictor groups. The lower row illustrates the averaged TSS difference among all SDMs when adding either spatial, temporal or both spatial and temporal (spatio temp.) predictors to SDMs based on mean predictors only.

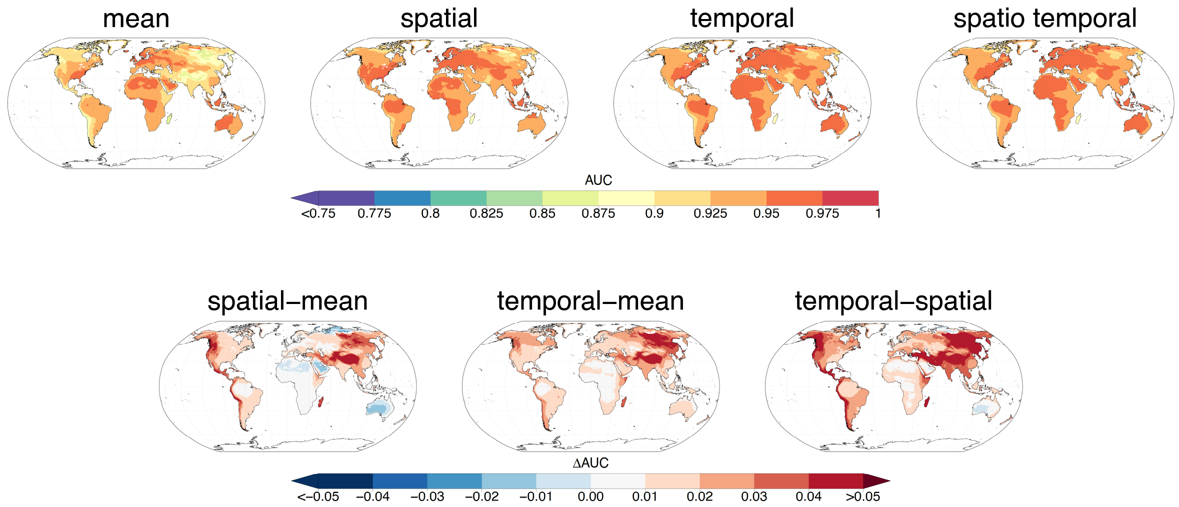

Supplementary Figure S2 Spatial variation in mean AUC (Area under the Curve) values per grid cell and AUC differences between models using different predictor groups. The upper row illustrates AUC averaged for all mammals, reptiles, and amphibians modeled for the four models using different predictor groups. The lower row illustrates the averaged AUC difference among all SDMs when adding either spatial, temporal or both spatial and temporal (spatio temp.) predictors to SDMs based on mean predictors only.

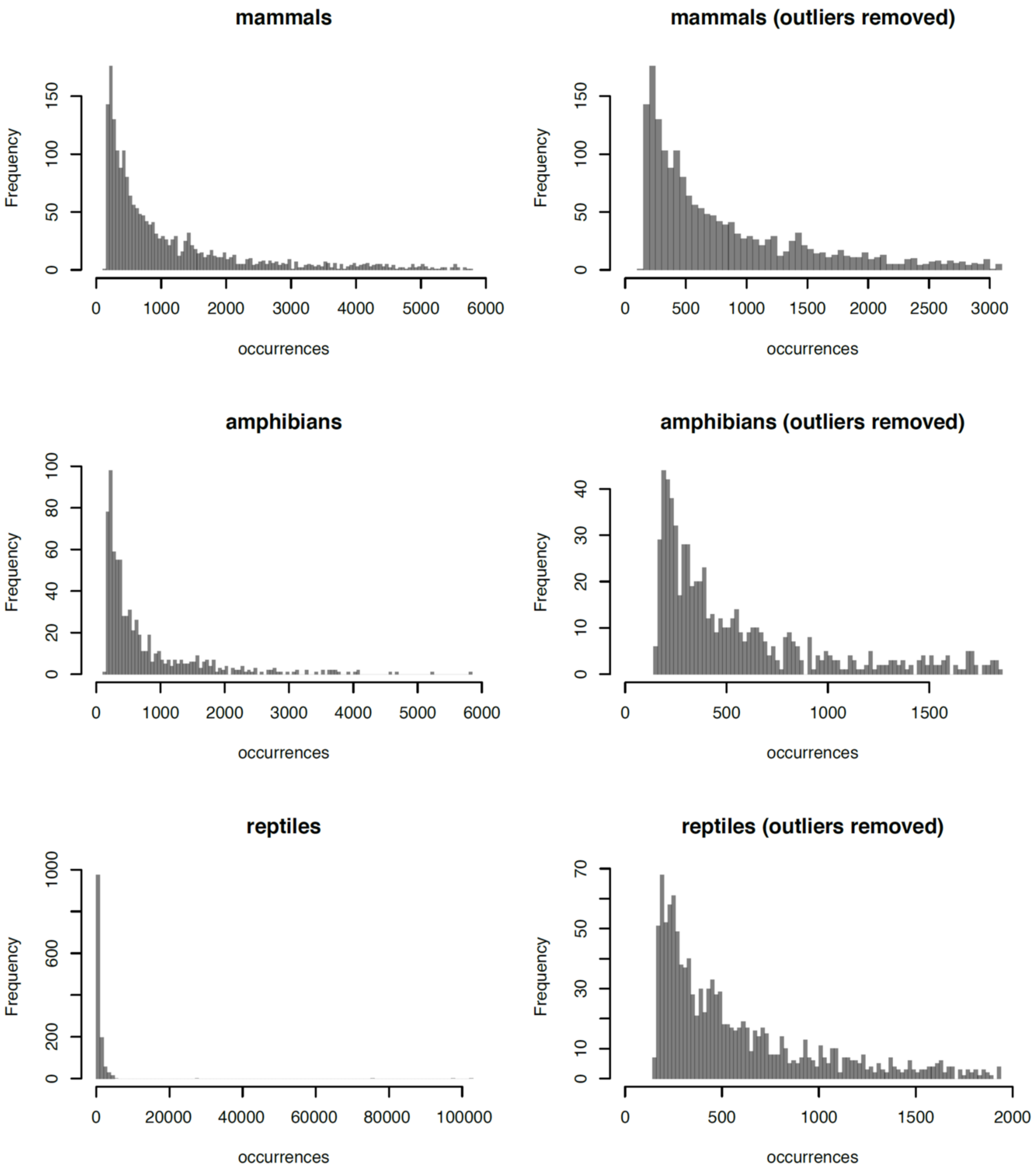

Supplementary Figure S3 Frequency of occurrences for all species from the three groups with the full dataset shown (left), and after the removal of outliers (right) for better readability. Occurrences are given as the number of 0.5° pixels occupied per species. Outliers were defined as those values that are within the 0.75 quantile.

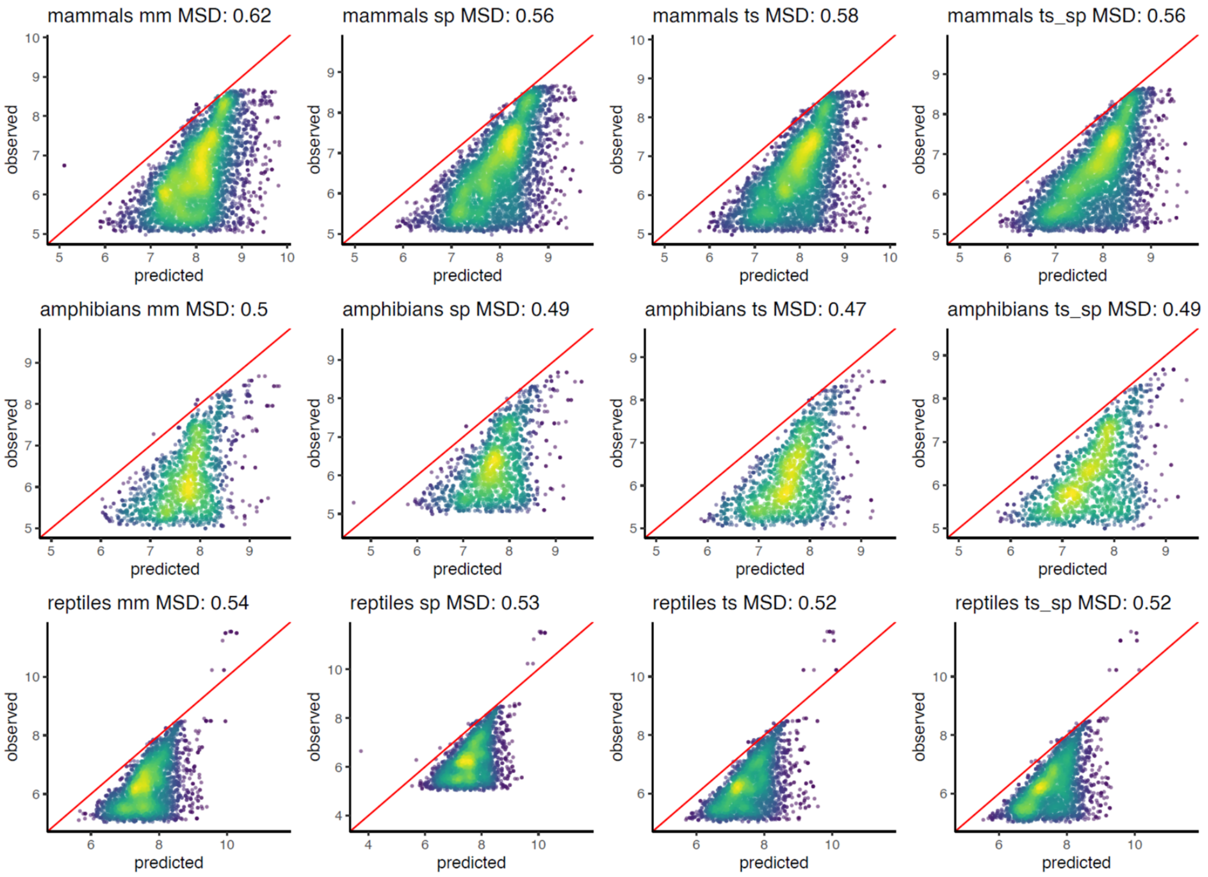

Supplementary Figure S4 Comparison of observed (from IUCN range maps) and predicted (ensemble mean from all three SDMs) range sizes for all three groups. mm = range sizes from SDMs using only the mean climate predictor group, sp = range sizes from SDMs using the mean climate predictor group and the spatial group, ts = range sizes from SDMs using the mean climate predictor group and the temporal group, ts_sp = range sizes from SDMs using the mean climate predictor group and the spatio-temporal group, MSD = mean squared deviation.

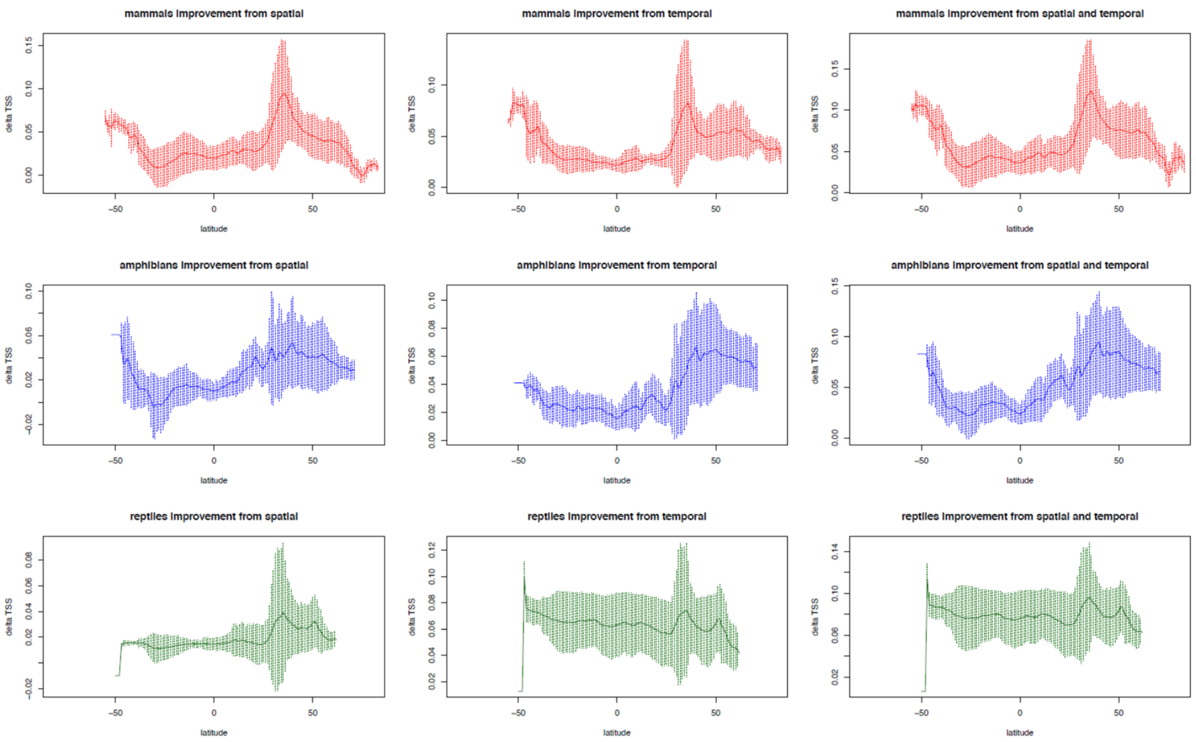

Supplementary Figure S5 Improvement in TSS (delta TSS) with latitude calculated by SDMs using the mean climate predictor group with SDMs the spatial group (left), the temporal group (middle) and the spatio-temporal group (right) for mammals (red, top row), amphibians (blue, middle row), and reptiles (green, bottom row). The solid line represents the mean delta TSS in 1° latitudinal bands, and the shaded area indictates the standard deviation of the delta TSS in 1° latitudinal band.

Supplementary Table S1 ODMAP protocol.

section	subsection	element	Value
Overview	Authorship	Study title	Interannual climate variability data improves niche estimates in species distribution models
Overview	Authorship	Author names	Dirk Nikolaus Karger[1], Bianca Saladin, Rafael O. Wüest, Catherine H. Graham, Damaris Zurell, Lidong Mo, and Niklaus E. Zimmermann
Overview	Authorship	Contact	dirk.karger@wsl.ch
Overview	Model objective	Model objective	Mapping and interpolation
Overview	Model objective	Target output	habitat suitability, suitable vs. unsuitable habitat
Overview	Focal Taxon	Focal Taxon	amphibians, reptiles, mammals
Overview	Location	Location	global
Overview	Scale of Analysis	Spatial extent	0, 180, -90, 90 (xmin, xmax, ymin, ymax)
Overview	Scale of Analysis	Spatial resolution	50
Overview	Scale of Analysis	Temporal extent	1979-2013
Overview	Scale of Analysis	Temporal resolution	35 years
Overview	Scale of Analysis	Boundary	natural
Overview	Biodiversity data	Observation type	range map
Overview	Biodiversity data	Response data type	presence/absence
Overview	Predictors	Predictor types	climatic
Overview	Hypotheses	Hypotheses	Spatial and temporal variability of climate are important predictors of species range limits.
Overview	Assumptions	Model assumptions	Species-environment equilibrium. No observation bias issues.
Overview	Algorithms	Modelling techniques	glm
Overview	Algorithms	Model complexity	We fitted models of medium complexity to account for complex non-linear species-climate relationships while reducing the risk of overestimation.
Overview	Algorithms	Model averaging	No ensembles were used.
Overview	Workflow	Model workflow	SDMs were fit to four different predictor sets (climate mean, climate mean + spatial variability, climate mean + temporal variability, climate mean + spatial and temporal variability). To assess model performance, we tested SDM predictions only within a 3000km buffer around each speciesâ€™ range polygon. We evaluated the predictive performance of the SDMs using repeated 80-20 split-sample tests (n=30). Predictive performance was assessed using the true skills statistic (TSS), after thresholding the predictions into presence/absence using a TSS-optimized threshold, and the area under the curve (AUC).
Overview	Software	Software	R version 3.6.1. R packages: raster, mgcv, randomForest
Overview	Software	Code availability	https://gitlabext.wsl.ch/karger/climate_variability_sdms
Overview	Software	Data availability	doi:10.16904/envidat.354

Data	Biodiversity data	Taxon names	730 amphibian, 1276 reptile, 1961 mammal
Data	Biodiversity data	Taxonomic reference system	IUCN
Data	Biodiversity data	Ecological level	species
Data	Biodiversity data	Data sources	IUCN Red List of Threatened Species (https://www.iucnredlist.org/resources/spatial-data-download)
Data	Biodiversity data	Sampling design	expert-based
Data	Biodiversity data	Sample size	n>=72
Data	Biodiversity data	Clipping	none
Data	Biodiversity data	Scaling	Range maps were rasterised at 0.5° spatial resolution. Grid cells intersecting with a range map polygon were assigned as presence cells.
Data	Biodiversity data	Cleaning	We only considered species for which the presences cover at least 72 0.5° grid cells so that a minimum of six data points per predictor variable (including quadratic terms) was available for model building. We also removed domestic and aquatic species.
Data	Biodiversity data	Absence data	NA
Data	Biodiversity data	Background data	NA
Data	Biodiversity data	Errors and biases	NA
Data	Data partitioning	Training data	NA
Data	Data partitioning	Validation data	80-20 split-sample approach with 30 repititions.
Data	Data partitioning	Test data	NA
Data	Predictor variables	Predictor variables	mean annual 2m air temperature 1979-2013, mean annual precipitation sum 1979-2013, spatial standard deviation mean annual 2m air temperature 1979-2013 across all 0.0083334° grid cells overlapping with a 0.5Â° grid cell, spatial standard deviation mean annual precipitation sum 1979-2013 across all 0.0083334° grid cells overlapping with a 0.5° grid cell, temporal standard deviation mean annual 2m air temperature 1979-2013 across all years from 1979-2013, temporal coefficient of variation mean annual precipitation sum 1979-2013 across all years from 1979-2013,
Data	Predictor variables	Data sources	CHELSA V1.2: chelsa-climate.org/
Data	Predictor variables	Spatial extent	0, 180, -90, 90 (xmin, xmax, ymin, ymax)
Data	Predictor variables	Spatial resolution	0.0083334°
Data	Predictor variables	Coordinate reference system	Geographic Coordinate system
Data	Predictor variables	Temporal extent	1979-2013
Data	Predictor variables	Temporal resolution	1 year

Data	Predictor variables	Data processing	To calculate sub-grid heterogeneity of a climatic variable (hereafter: spatial) within a 0.5° grid cell, we used the standard deviation of all CHELSA 30 arc second grid cells overlapping with 0.5° grid cells. To calculate the interannual variability (hereafter: temporal) we calculated the standard deviation of mean annual 2m air temperature for each year from 1979 to 2013 from CHELSA V1.2 per grid cell. For temporal precipitation variability we used the relative standard deviation (temporal RSD, equivalent to the coefficient of variation) of the annual precipitation sum across all years from 1979 to 2013 from CHELSA V1.2 per grid cell.
Data	Predictor variables	Errors and biases	NA
Data	Predictor variables	Dimension reduction	NA
Data	Transfer data	Data sources	NA
Data	Transfer data	Spatial extent	global
Data	Transfer data	Spatial resolution	0.0083334
Data	Transfer data	Temporal extent	1979-2013
Data	Transfer data	Temporal resolution	normals
Data	Transfer data	Models and scenarios	NA
Data	Transfer data	Data processing	NA
Data	Transfer data	Quantification of Novelty	NA
Model	Variable pre-selection	Variable pre-selection	We heuristically selected mean annual temperature and annual precipitation as important determinants of large-scale species distributions.
Model	Multicollinearity	Multicollinearity	NA
Model	Model settings	Model settings (fitting)	glm: family (binomial), formula (linear + quadratic), weights (sum of weights of presences equaled the sum of weights of absences)
Model	Model settings	Model settings (extrapolation)	NA
Model	Model estimates	Coefficients	NA
Model	Model estimates	Parameter uncertainty	NA
Model	Model estimates	Variable importance	NA
Model	Model selection - model averaging - ensembles	Model selection	NA
Model	Model selection - model averaging - ensembles	Model averaging	NA

Model	Model selection - model averaging - ensembles	Model ensembles	NA
Model	Analysis and Correction of non-independence	Spatial autocorrelation	NA
Model	Analysis and Correction of non-independence	Temporal autocorrelation	NA
Model	Analysis and Correction of non-independence	Nested data	NA
Model	Threshold selection	Threshold selection	TSS-optimizing threshold
Assessment	Performance statistics	Performance on training data	NA
Assessment	Performance statistics	Performance on validation data	AUC
Assessment	Performance statistics	Performance on test data	NA
Assessment	Plausibility check	Response shapes	NA
Assessment	Plausibility check	Expert judgement	NA
Prediction	Prediction output	Prediction unit	habitat suitability, predicted presence/absence
Prediction	Prediction output	Post-processing	NA
Prediction	Uncertainty quantification	Algorithmic uncertainty	NA
Prediction	Uncertainty quantification	Input data uncertainty	We assessed how SDM performance varied across climate predictor sets including or excluding spatial and temporal variability.
Prediction	Uncertainty quantification	Parameter uncertainty	NA
Prediction	Uncertainty quantification	Scenario uncertainty	NA
Prediction	Uncertainty quantification	Novel environments	NA
